# Treatment of multisegmental vertebral compression, burst fractures, and sandwich vertebra with severe osteoporosis using the PKP technique: a case report and literature review

**DOI:** 10.3389/fneur.2023.1118891

**Published:** 2023-09-06

**Authors:** Bo Han, Daming Pang, Yong Hai, Jincai Yang, Zhexuan Fan, Haifeng Gao, Peng Yin

**Affiliations:** Department of Orthopedics, Affiliated Beijing Chaoyang Hospital of Capital Medical University, Beijing, China

**Keywords:** sandwich vertebra, multisegmental vertebral compression fracture, burst fracture, percutaneous kyphoplasty, osteoporosis

## Abstract

This study aimed to present a special case of treatment of a patient with multisegmental vertebral compression fracture, burst fracture, and sandwich vertebra and to review the literature on this condition. An 85 year-old female presented with severe low back pain but no radiating pain in the lower extremities. The patient was diagnosed with T12 and L5 vertebral compression fractures, fresh vertebral burst fractures in L2 and L3, and osteoporosis. The focus was on formulating a surgical treatment strategy. At the 12 month follow-up, no neurological deficits were observed, and the chosen surgical treatment approach yielded favorable clinical outcomes. A comprehensive literature review indicates that percutaneous kyphoplasty (PKP) can effectively alleviate pain and ensure safety in managing osteoporotic vertebral burst fractures. While complications remain a theoretical risk, they can be mitigated through meticulous assessment, careful surgical procedures, and appropriate preventive measures. PKP is an effective and safe treatment modality for osteoporotic vertebral burst fractures. Conservative management of sandwich vertebrae can yield positive clinical outcomes, but regular anti-osteoporosis treatment is necessary.

## Background

Osteoporosis is a bone disease characterized by impaired bone strength, which increases the likelihood of fracture (1). Over the past several decades, the incidence of osteoporosis has increased with the rapid increase in the proportion of older adults globally. Osteoporotic fractures are a serious problem. Specifically, spinal fractures are a common type of osteoporotic fracture that seriously affect the quality of life of older adults (2).

However, the treatment of osteoporotic vertebral burst fractures has not been unified yet. Conservative treatment is often not available because of the high risk of secondary nerve injury caused by pain, limited movement, and local instability (3, 4). Traditional open surgery, such as short- or long-segment pedicle screw fixation, has achieved satisfactory results in nonosteoporotic patients with high-energy trauma (5). However, there is usually a high incidence of complications in patients with osteoporosis, such as screw loosening, displacement, or postoperative systemic complications (6). In recent years, percutaneous kyphoplasty (PKP) has been widely used to treat osteoporotic compression fractures and has achieved good clinical results (7). Previous studies reported that a decrease in the height of the anterior and posterior edges of the vertebral body, fracture of the posterior wall of the vertebral body, and cortical defects of the vertebral body are risk factors for bone cement leakage in osteoporotic vertebral burst fractures. In addition, some researchers believe that PKP can easily shift part of the fracture into the spinal canal and cause cement leakage along the posterior wall of the ruptured vertebral body (8). Therefore, osteoporotic vertebral burst fractures represent contraindications for percutaneous kyphoplasty. However, recent studies have shown that PKP can achieve satisfactory clinical results without postoperative complications (9).

In addition, a randomized controlled trial favored vertebral augmentation over conservative treatment for symptomatic osteoporotic vertebral compression fractures because vertebral augmentation can relieve back pain and strengthen the fractured vertebrae (7). Although percutaneous vertebroplasty is effective in the treatment of symptomatic osteoporotic vertebral compression fractures, previous studies have reported that the incidence of new osteoporotic fractures in adjacent segments after vertebral augmentation is approximately 6.3–47.5% (10, 11). This technique increases the stiffness and strength of the augmented segment, resulting in changes in the load distribution on the adjacent vertebrae. A sandwich vertebral body (SVB) was defined as an intact unaugmented vertebral body between two previously augmented vertebrae (12). With the double-load shift, increased stiffness and strength at the adjacent segment, and pre-existing severe osteoporosis, the SVB may increase the risk of developing new vertebral fractures. However, some researchers believe that changes in the biomechanical indices of sandwich vertebrae can be ignored (13). Furthermore, regarding the treatment of sandwich vertebrae, some researchers have suggested prophylactic vertebral augmentation (14), but others have concluded that there is no significant difference in the refracture rate of sandwich vertebrae between the two groups by comparing the conservative treatment of sandwich vertebrae with bone cement-reinforced sandwich vertebrae (15). Whether the high incidence of new adjacent segmental vertebral fractures is related to vertebral augmentation and how to manage sandwich vertebrae are debated.

The treatment of patients with multisegmental vertebral compression fractures, burst fractures, and sandwich vertebrae has not been reported. This report discusses a patient with these conditions and provides a treatment protocol.

## Case presentation

An 85 year-old female visited our clinic because of severe low back pain after experiencing an accidental fall 8 days prior. Lower back pain was obvious when turning over and getting out of bed. The patient’s symptoms improved after the patient was lying down. The symptoms were localized over the lower back without lower extremity radiation pain. The VAS score for low back pain was 9, and the ODI score was 80%. During the patient’s physical examination, we noted that lumbar flexion and extension activities were restricted, as well as severe slamming pains at the T12, L2, and L3 spinous process levels. Muscle strength and sensation in the lower limbs were normal. The bilateral deep tendon and ankle reflexes were normal. Results of the bilateral straight-leg raising test, tension test of the femoral nerve, and Babinski sign test were negative.

**Figure 1:**
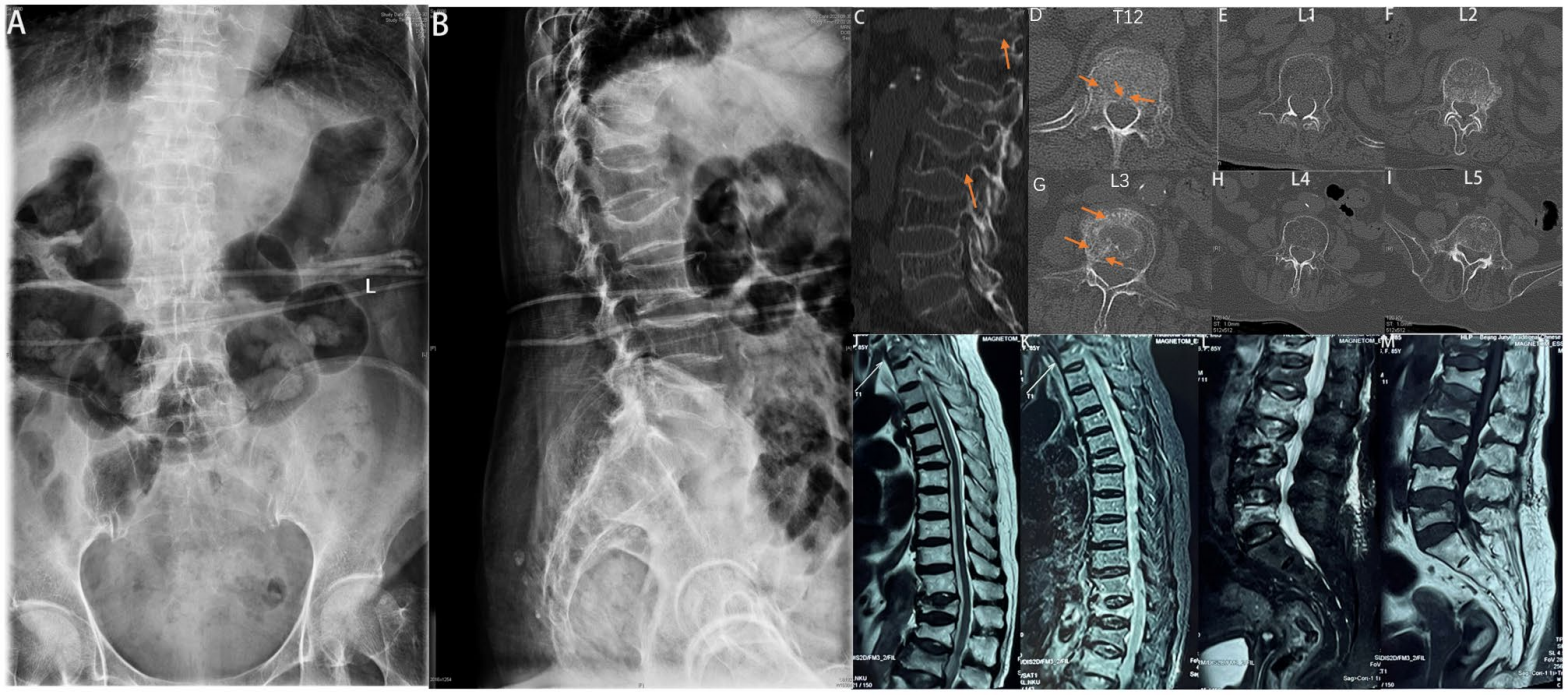
Preoperative radiograph of the spine. **(A)** Anteroposterior and **(B)** lateral radiographs showing T12 and L5 vertebral compression fractures and L2 and L3 vertebral burst fractures. Preoperative CT of the spine **(C−I)**. T12 and L5 vertebral compression fractures, and L2 and L3 vertebral burst fractures. Preoperative MRI of the spine **(J−M)**. T12 and L5 vertebral compression fractures, and L2 and L3 vertebral burst fractures.

Preoperative radiographs, computed tomography (CT), and magnetic resonance imaging (MRI) examination showed T12 and L5 fresh vertebral compression fracture and L2, L3 fresh vertebral burst fracture (Figure 1). The bone density and value were both −3.2. The patient was diagnosed with T12 and L5 vertebral compression fractures, L2 and L3 fresh vertebral burst fractures, and osteoporosis.

**Figure 2:**
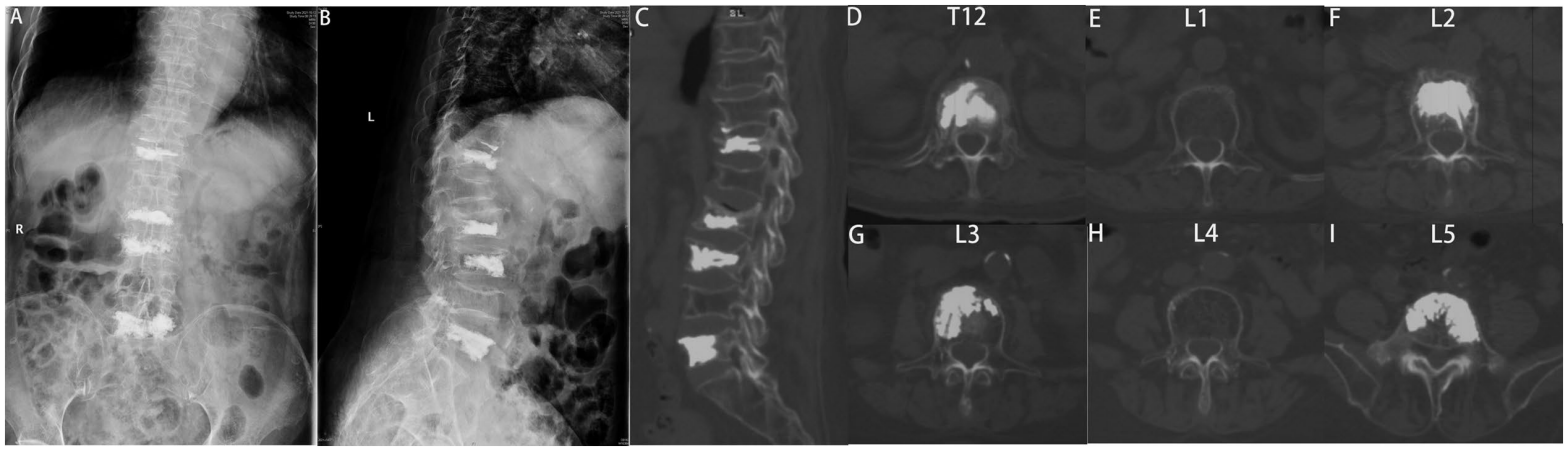
Postoperative X-rays of the spine. **(A)** Anterior–posterior and **(B)** lateral radiographs demonstrating successful bone cement filling without any leakage. The final follow-up CT scan of the spine **(C−I)** showing no evidence of new fractures.

After discussing the surgical strategy, including anesthesia method, surgical stage, intraoperative risks, and complications, we performed stage one multisegmental PKP (T12, L2, L3 unilateral PKP, and L5 bilateral PKP) (Figure 2). The puncture operation of the T12 and L3 unilateral PKP was carried out simultaneously. In a prone position, the patient was administered local anesthesia. The needle was then carefully inserted into the vertebrae under C-armed fluoroscopic guidance. During this process, the needle should avoid over-proximity to the cortical defects, especially away from the posterior wall defects. We carefully performed balloon inflation and high-viscosity cement injections under fluoroscopy. The starting time of cement injection was approximately 4 min.

The operative time was 75.0 min, the fluoroscopy time was 6 min, and there was no cement leakage. The VAS score for lower back pain was 3.0, and the ODI score was 15.0%. The patients were advised to wear suitable braces for at least 1 month and to receive anti-osteoporosis therapy regularly after the operation. The patient’s preoperative symptoms significantly improved. Postoperative CT showed no cement leakage, and the bone cement was sufficiently distributed (Figure 2). No surgical complications, including pedicle perforation, cement leakage, neurovascular injury, or infection, were observed during the 12 month follow-up period.

After the operation, the patient received regular anti-osteoporotic therapy (calcium carbonate, calcitriol, and denosumab), wore a brace for over 3 months, and avoided bending down and weight-bearing.

## Discussion and conclusion

For patients with multisegmental fractures, including L2 and L3 osteoporotic burst fractures, as well as T12 and L5 osteoporotic compression fractures, we employed PKP. Specifically, unilateral PKP was performed for T12, L2, and L3, while bilateral PKP was performed for L5. However, preventive surgery was not conducted for the sandwich vertebrae at L1 and L4. After a 12 month follow-up, significant clinical improvements were observed. The visual analog scale (VAS) score for low back pain was 1, indicating minimal pain, and the Oswestry Disability Index (ODI) score was 10%, reflecting a low level of disability. Additionally, no new fractures were detected during the follow-up period.

PKP has been proven effective and safe for the treatment of osteoporotic vertebral compression fractures (16). However, the treatment of osteoporotic vertebral burst fractures with PKP remains controversial. Conservative treatment often causes secondary nerve injury, aggravation of kyphosis, pseudoarthrosis, and other complications (3). Traditional surgical techniques such as pedicle fixation have been successfully implemented in previous studies to treat burst fractures (17). However, a high failure rate of pedicle fixation, such as loosening of internal fixation and displacement, is not uncommon in patients with osteoporosis. In addition, older adults with osteoporosis who experience persistent pain or chronic diseases may not tolerate such surgery (6). PKP is a minimally invasive procedure involving less bleeding and a shorter lying time and is more suitable for osteoporosis patients with persistent pain or chronic diseases (7). To fully understand the role of PKP in the treatment of osteoporotic vertebral burst fractures and evaluate its efficacy, we summarized 10 related cohort studies conducted over the past 30 years (Table 1) (9, 18–26).

**Table 1:** Summary of study characteristics of included trials.

Studies	Region	Study design	Number of patients	Age (years)	Gender M/F	Follow-up (months)
He D	China	Prospective RCT	22	73.18 ± 4.90	11/11	34.00 ± 9.41 (24–59)
Kruger A	Germany	Retrospective Cohort	97	76.1 ± 12.36	29/68	20.2 ± 9.79 (5–48)
An KC	Korean	Retrospective Cohort	12	78 (66–84)	0/12	≥12
Stoffel M	Germany	Prospective Cohort	74	72 (34–95)	22/52	15 (8–32)
Fuentes S	France	Prospective Cohort	18	53 (22–78)	12/6	26 (17–30)
Zhang L	China	Retrospective Cohort	23	63.7 ± 5.8 (58–72)	7/16	24
Yin P	China	Prospective Cohort	46	75.9 ± 7.6 (55–88)	18/28	28.8 ± 7.0
Gan M	China	Prospective Cohort	25	69 (56–82)	7/18	≥6
Li YM	China	Retrospective Cohort	111	71.48 ± 7.01	25/86	24
Tang CG	China	Prospective Cohort	68	71.37 ± 7.04	16/46	24

All studies reported VAS scores, and five studies assessed ODI scores (Table 2) (9, 20, 23, 25, 26). The results showed that PKP could relieve pain and is safe for treating osteoporotic thoracolumbar burst fractures. Compared to short-segment pedicle internal fixation, PKP can quickly relieve pain and reduce the hospital stay. Five studies evaluated vertebral height correction based on the height of the anterior and posterior edges of the vertebral body (9, 19, 20, 25, 26), and all 10 studies observed perioperative kyphosis. The results showed that the kyphosis angle significantly improved after PKP. Gan et al. explored the feasibility and clinical results of PKP for treating osteoporotic thoracolumbar burst fractures in patients without neurological disorders. They concluded that this technique could reduce pain and increase vertebral body height without worsening it (20). Furthermore, Zhang et al. compared the clinical outcomes of PKP and short-segment pedicle fixation. The results showed that these two approaches are effective surgical methods for treating thoracolumbar burst fractures within a short time (26). Notably, PKP significantly reduces blood loss and bed rest time. Fuentes et al. conducted a prospective study evaluating the efficacy of PKP and pedicle screw fixation for treating thoracolumbar burst fractures (19). The results showed that this approach’s vertebral height recovery and kyphosis correction rates were similar to those of open surgery and may be an alternative to open surgery.

**Table 2:** Clinical and radiological outcomes and complications of percutaneous kyphoplasty.

Results		He D	Kruger A	An KC	Stoffel M	Fuentes S	Zhang L	Yin P	Gan M	Li YM	Tang CG
Number of vertebrae		22	110	13	81	18	23	46	25	111	62
VAS	pre-	8	8.1 ± 0 0.815	8.3 ± 0.4	7.0 ± 0.3	6.8 (4–8)	8.0 ± 1.0	8.2 ± 0.7	8.2 ± 0.7	7.04 ± 1.15	7 (6–8)
	post-	3	/	3.9 ± 0.2	2.3 ± 0.2	1.1 (0–2)	2.8 ± 0.7	1.8 ± 0.7	2.8 ± 0.8	2.27 ± 1.04	2 (1–3)
	final	2	1.6 ± 1.02	3.1 ± 0.17	/	1.1 (0–2)	2.3 ± 0.6	0.7 ± 0.7	2.9 ± 1.1	1.87 ± 0.84	2 (1–2)
ODI	pre-	/	/	/	/	/	68.4 ± 8.9%	87.0 ± 6.0%	68.2 ± 6.6%	67.11 ± 13.49	71.40 ± 13.52
	post-	/	/	/	/	/	34.2 ± 3.2%	23.9 ± 4.4%	35. 3 ± 2.8%	22.00 ± 11.20	21.78 ± 11.21
	final	/	/	/	/	/	33.9 ± 5.1%	19.1 ± 3.8%	34.5 ± 1.8%	16.18 ± 9.11	16.02 ± 7.76
Complications	Cement leakage	10	46	1	17	2	4	8	4	31	10
	Adjacent fracture	3	5	/	6	/	/	2	/	/	/
Kyphotic angle (KA)	pre-	11°	8.53° ± 6.3 (−5 to 27°)	15.9 ± 2.4°	10 ± 1°	14.44° (5 to 35°)	16.9 ± 9.1°	17.9 ± 1.4	21.7 ± 7.8	13.33 ± 4.26	11.88 ± 4.28
	Post-	7°	4.77° ± 3.97°(−2 to 14°)	6.2 ± 1.6°	5 ± 1°	3.17° (−5 to 10°)	11.9 ± 7.9°	14.2 ± 1.9	8.6 ± 6.6	10.04 ± 4.26	8.99 ± 4.06
Height of anterior vertebra (Ha)	pre-	/	/	/	/	65% (36–83%)	64.1 ± 14.8%	20.1 ± 2.3	61.5 ± 13.9	65.10 ± 10.54%	65.69 ± 10.51
	Post-	/	/	/	/	89% (67–100%)	80.7 ± 12%	22.9 ± 2.4	85.3 ± 10.6	81.04 ± 10.18%	81.10 ± 11.78
Height of posterior vertebra (Hp)	pre-	/	0.808 ± 0.182	/	/	65% (40–83%)	87 ± 8.7%	27.3 ± 1.7	73.0 ± 9.3	/	86.69 ± 6.78
	Post-	/	0.875 ± 0.118	/	/	92% (82–100%)	92.2 ± 6.0%	28.1 ± 1.7	83.3 ± 7.4	/	91.43 ± 6.71

In addition, all the included studies reported postoperative complications. Bone cement leakage was the most common complication, ranging from 7.7 to 45.4% in ten studies; however, no neurological symptoms were observed. Leakage of bone cement may lead to serious consequences such as embolism of distant organs or oppressive symptoms. However, Stoffel et al. believed that the potential surgery-related complications caused by the posterior wall displacement of the vertebral body seemed to be a theoretical risk rather than an actual risk in burst fractures (24). Moreover, Walter et al. conducted a meta-analysis to evaluate the frequency and pattern of bone cement leakage in vertebra burst fractures and osteoporotic compression fractures (27). They concluded that PKP could be a safe treatment method for burst fractures. In addition, the longitudinal ligaments and soft tissue around the vertebral body can potentially prevent bone–cement leakage. Lastly, Yin et al. mentioned that cement leakage could be prevented through careful assessment, careful operation, and appropriate preventive measures (9).

Management of sandwich vertebrae, in this case, was also critical. The sandwich vertebra is a completely unfractured vertebral body between two augmented vertebrae. There is great controversy regarding the long-term prognosis of sandwich vertebrae and the risk of refracture. Some researchers believe that sandwich vertebrae’s upper and lower endplates can withstand additional stress from the augmented vertebrae. A biomechanical study on human cadavers showed that the stress and biomechanical indices of sandwich vertebrae’s endplate and intervertebral space changed (28). However, another study reached the opposite conclusion, suggesting that changes in the biomechanical properties of sandwich vertebrae are almost negligible after vertebral augmentation (13). Tang et al. (14) performed preventive surgery for sandwich vertebrae and reported long-term follow-up results. Their study suggested that preventive vertebral augmentation with bone cement could reduce the incidence of sandwich vertebral fractures. However, Yang et al. focused on the fate of the sandwich vertebra and the treatment strategy. Their study compared the incidence of sandwich vertebral refractures after vertebral augmentation and conservative treatment. In that study, there was no significant difference in the incidence of sandwich vertebral fractures between the surgical and conservative groups (29). A retrospective study in 2021 that aimed to determine the incidence of refracture in the sandwich vertebra compared with the adjacent segment vertebra showed that patients with sandwich vertebral bodies had a relatively high risk of developing new fractures after vertebral augmentation compared with previous studies (12). However, there was no statistically significant difference in the incidence of new fractures between the sandwich and adjacent vertebral body groups. This study also suggests that severe osteoporosis is a major risk factor for an increased incidence of refractures. Based on the aforementioned studies, the patient is highly recommended to undergo regular anti-osteoporosis therapy, despite the absence of preventive surgery for the sandwich vertebra. The 12 month follow-up assessment revealed no incidence of new fractures (31).

Mmulti-stage PKP surgery could be performed under general anesthesia (30). However, the reason we chose to conduct the surgery under local anesthesia is specific to our case. We had thorough discussions and planning regarding the choice of anesthesia prior to the procedure. Firstly, the patient is an 85 year-old elderly female with multiple underlying conditions and obesity. Given these factors, we consider that the use of a general anesthesia approach would pose relatively higher risks (31). Secondly, the patient’s spinal CT imaging and bone density indicate severe osteoporosis. In cases of severe osteoporosis, there is typically lower resistance during instrument insertion and balloon dilation compared to normal patients, making the procedure easier to complete in a shorter time frame (32). Additionally, after communicating with the patient, the patient had a strong hospital desire to try local anesthesia and fully agreed to change the anesthesia regimen when it was intolerable. After weighing all pros and cons, we chose local anesthesia while keeping general anesthesia as an alternative. Throughout the procedure on the elderly patient, we maintained constant communication and interaction. Every 10 min, we inquired about the VAS pain score for the surgical area (33). While providing humane care, we simultaneously evaluated the patient’s tolerance to pain. If the patient becomes unable to tolerate local anesthesia, we would promptly switch to general anesthesia (12).

The treatment duration of a staged operation is long, and multiple punctures increase the patient’s psychological, economic, and surgical risks. We cshose stage I surgical treatment to reduce the patients’ fear and resistance to the operation, operation time, amount of intraoperative blood loss, and the number of fluoroscopies. We chose T12, L2, and L3 unilateral punctures and L5 bilateral punctures to ensure the symmetrical distribution of bone cement in the L5 vertebral body and to stabilize the L5 vertebral body. According to the study, although the incidence of operative complications may increase when more than three target vertebrae are undergoing a Stage I operation, complications can be prevented and reduced by shortening the operation time, reducing the amount of bone cement injected, choosing reasonable surgical techniques, and implementing corresponding preventive measures. No new fractures were found immediately after surgery or at 12 months follow-up.

This study had some limitations. First, the follow-up period was short, and long-term postoperative complications were not observed. Moreover, a large number of subjects and further studies are needed to evaluate the clinical outcomes.

PKP is an effective and safe treatment option for osteoporotic vertebral burst fractures. Conservative treatment of sandwich vertebrae can achieve good clinical outcomes, and regular anti-osteoporosis treatment is required. Through StageImulti-segmental PKP (T12, L2, L3 unilateral PKP, and L5 bilateral PKP), the operation time is effectively shortened, the number of fluoroscopies is reduced, and the economic and psychological burden of patients is reduced.

## Data availability statement

The raw data supporting the conclusions of this article will be made available by the authors, without undue reservation.

## Ethics statement

The studies involving humans were approved by Ethics Committee of Beijing Chaoyang Hospital affiliated to Capital Medical University. The studies were conducted in accordance with the local legislation and institutional requirements. The participants provided their written informed consent to participate in this study. Written informed consent was obtained from the participant/patient(s) for the publication of this case report.

## Author contributions

PY, YH, BH, and DP contributed to the operation and the design of the study. JY, HG, and ZF were in charge of data collection. DP, BH, and HG contributed to the information of follow-up. BH and DP contributed to the pictures’ adjustment and statistical analysis. All authors contributed to the article and approved the submitted version.

## Conflict of interest

The authors declare that the research was conducted in the absence of any commercial or financial relationships that could be construed as a potential conflict of interest.

## Publisher’s note

All claims expressed in this article are solely those of the authors and do not necessarily represent those of their affiliated organizations, or those of the publisher, the editors and the reviewers. Any product that may be evaluated in this article, or claim that may be made by its manufacturer, is not guaranteed or endorsed by the publisher.

## Abbreviations

VAS-LBP: Visual analog scale on lumbar pain
VAS-LP: Visual analog scale on leg pain
ODI: Oswestry Disability Index
PKP: Percutaneous kyphoplasty
SVB: Sandwich vertebral body
